# Silibinin Inhibits Cell Ferroptosis and Ferroptosis-Related Tissue Injuries

**DOI:** 10.3390/antiox12122119

**Published:** 2023-12-15

**Authors:** Wentao Duan, Zexian Ou, Yuxing Huang, Yifan Zhang, Lan Zhang, Yanan Zhao, Ruikun He, Yihan Zhang, Yuanlong Ge, Huiling Lou, Zhenyu Ju, Qian Hu

**Affiliations:** 1 Key Laboratory of Regenerative Medicine of Ministry of Education, Institute of Aging and Regenerative Medicine, College of Life Science and Technology, Jinan University, Guangzhou 510632, China; 200919961220.hi@163.com (W.D.); ouzexian@foxmail.com (Z.O.); huangyuxingi@163.com (Y.H.); mzhangyifanm@163.com (Y.Z.); 13650163810@163.com (L.Z.); ashelyzyn@outlook.com (Y.Z.); geyuanlong@jnu.edu.cn (Y.G.); 2 BYHEALTH Institute of Nutrition & Health, Guangzhou 510663, China; herk@by-health.com (R.H.); zhangyh7@by-health.com (Y.Z.); 3 Department of Geriatrics, National Key Clinical Specialty, Guangzhou First People’s Hospital, School of Medicine, South China University of Technology, Guangzhou 510180, China

**Keywords:** ferroptosis, silibinin, glutathione peroxidase 4, renal ischemia reperfusion

## Abstract

Ferroptosis is involved in various tissue injuries including neurodegeneration, ischemia-reperfusion injury, and acute liver injury. Ferroptosis inhibitors exhibit promising clinical potential in the treatment of various diseases. As a traditional chemical, silymarin has been widely used in healthcare and clinical applications to treat liver injuries in which ferroptosis is involved. Silibinin is the main active ingredient of silymarin. However, the effect of silibinin on ferroptosis and ferroptosis-related diseases remains unclear. Here, we found that silibinin inhibited death in different kinds of cells caused by ferroptosis inducers including RSL3 and erastin. Moreover, silibinin alleviated lipid peroxidation induced by RSL3 without affecting the labile iron pool. Next, the antioxidant activity of silibinin was demonstrated by the DPPH assay. In vivo, silibinin strikingly relieved tissue injuries and ferroptosis in the liver and kidney of glutathione peroxidase 4 (GPX4) knockout C57 BL/6J mice. Moreover, silibinin effectively rescued renal ischemia-reperfusion, a well-known ferroptosis-related disease. In conclusion, our study revealed that silibinin effectively inhibits cell ferroptosis and ferroptosis-related tissue injuries, implicating silibinin as a potential chemical to treat ferroptosis-related diseases.

## 1. Introduction

Ferroptosis is a form of regulated cell death, resulting from the iron-dependent accumulation of lipid peroxidation [1]. The accumulation of labile iron, the synthesis of polyunsaturated fatty acids (PUFA)-containing phospholipids, and the failure of the lipid peroxide repair network are key to ferroptosis [2]. Labile iron facilitates the oxidation of PUFA through the Fenton reaction and lipoxygenase [3]. Glutathione peroxidase 4 (GPX4), a critical member of the lipid peroxide repair network, prevents ferroptosis with glutathione (GSH) [4,5]. The depletion of GPX4 leads to cell ferroptosis [4]. Mice with the loss of GPX4 cannot survive during embryonic development [6]. Conditional GPX4-deficient mice present dysfunction in multiple tissues such as the liver, kidney, and hematopoietic system [4,7,8]. Physiologically, ferroptosis is involved in oxidative-stress-related injuries such as ischemia-reperfusion injury (IRI), acute liver and kidney injury, and neurodegenerative diseases [9].

Efficient ferroptosis inhibitors will bring unprecedented opportunities for the treatment of related diseases [10]. However, the known ferroptosis inhibitors such as iron chelators are limited by their off-target effect and toxicity, while the efficiency of hydrophobic antioxidants may vary widely in different contexts [11,12]. Silymarin, extracted from milk thistle, has long been used in the treatment of liver diseases with great safety and efficiency [13]. Silibinin (alternatively named silybin) is the main active component of silymarin [14]. As an effective antioxidant chemical, silibinin has been demonstrated to function with anti-inflammatory, protein-synthesis-enhancing, and anti-fibrotic activities [15,16]. Silibinin is widely used as a supportive drug or healthcare product in the treatment of acute and chronic liver diseases such as alcoholic and nonalcoholic fatty liver, liver cirrhosis, and drug-induced liver injury [17]. Interestingly, ferroptosis has been reported to be involved in these liver injuries [18]. However, the effects of silibinin on cell ferroptosis and ferroptosis-related diseases remain unclear.

In the current study, we showed that silibinin inhibits cell ferroptosis induced by either RSL3 or erastin in different cell lines. In addition, silibinin did not affect the labile iron pool in cells, and its ferroptosis inhibitory effect was mainly achieved by inhibiting lipid peroxidation through antioxidant ability. Then *Gpx4*^flox/flox^ ERT-Cre mice were used to induce systemic GPX4 deletion in multiple tissues. We found that silibinin effectively alleviated ferroptosis-related injury in the liver and kidney. Lastly, silibinin ameliorated renal IRI, in which ferroptosis plays a critical role. These results suggest the clinical potential of silibinin in ferroptosis-related diseases.

## 2. Materials and Methods

### 2.1. Mice

Two-month-old C57 BL/6J wild-type mice were provided by GemPharmatech (Nanjing, China). The *Gpx4*^flox/flox^ mouse was a gift from Professor Fudi Wang at Zhejiang University. ERT-Cre mice were crossed with *Gpx4*^flox/flox^ mice to generate the *Gpx4*^flox/flox^ ERT-Cre mice. Animals were randomly assigned to each group. *Gpx4*^flox/flox^ ERT-Cre mice were intraperitoneally injected with 100 mg/kg tamoxifen three times every other day to obtain GPX4-deficient (accession number NC_000076.7) mice. At the same time, *Gpx4*^−/−^ + sili mice were treated with silibinin (100 mg/kg) through gavage every day until they were sacrificed 12 days later. The study was conducted according to the guidelines of the Declaration of Helsinki. All experiments were approved by the Animal Protection and Ethics Committee of Jinan University (101194, 20230320).

### 2.2. Cell Culture

HT1080 is a human fibrosarcoma cell line, HepG2 is a human hepatoma cell line, BJ is a human skin fibroblast cell line, and 293T is a human embryonic kidney cell line. All cells were from Dr. Yong Zhao’ lab in Sun Yat-sen University. Cells were cultured with DMEM medium (Gibco, Waltham, MA, USA) supplemented with 10% fetal bovine serum (Gibco) and 1% penicillin/streptomycin (Gibco). Cells were cultured at 37 °C in a cell incubator with 5% CO_2_.

### 2.3. Chemicals

RSL3, erastin, α-Tocopherol (α-Toc), and Liproxstatin-1 (Lip-1) were purchased from Selleck, Houston, TX, USA. Deferoxamine (DFO) was purchased from MCE, Zelienople, PA, USA. Silibinin was purchased from TargetMol, Boston, MA, USA. Tamoxifen and ammonium iron(Ⅲ) citrate (FAC) were purchased from Sigma, Ronkonkoma, NY, USA.

### 2.4. Cell Viability Assay

Cells were plated in 96-well plates with 4000 cells (for HT1080, HepG2, or 293T) or 3000 cells (for BJ) per well and cultured overnight followed by the addition of indicated chemicals. HT1080 cells were stained with Hoechst 33342 (Sangon Biotech, Shanghai, China) and PI (Sangon Biotech) after 12 h (for RSL3) or 24 h (for erastin) treatment at 37 °C for 15 min. HepG2, 293T, and BJ cells were stained with Hoechst 33342 (Sangon Biotech) and PI (Sangon Biotech) after 24 h (for erastin or RSL3) treatment at 37 °C for 15 min. Then, the cell images were captured with ImageXpress Micro Confocal (Molecular Devices, San Jose, CA, USA), and cell viability was measured with MetaXpress version 6.6.1.42 (Molecular Devices).

### 2.5. Lipid Peroxidation Measurement

Cells were plated in 6-well plates. When reaching 80% confluency, cells were treated with drugs for 4 h. After being digested by trypsin and washed with PBS, cells were stained with 5 μM C11-BODIPY (Life Technology, Carlsbad, CA, USA) in PBS for 30 min at 37 °C. After PBS washing, the cells were suspended in PBS and applied for flow cytometry. Lipid ROS levels were measured with the FITC channel by Fortessa (BD, Franklin Lakes, NJ, USA).

### 2.6. Labile Iron Pool Measurement

Cells were plated in 12-well plates. When reaching 80% confluency, cells were treated with drugs for 24 h and then were digested with trypsin. After PBS washing, cells were then stained with Calcein-AM (0.25 μM) for 10 min at 37 °C. After PBS washing, the cells were suspended in PBS. Labile iron pools were measured using the FITC channel by flow cytometry with Fortessa (BD).

### 2.7. Prussian Blue Staining

After being treated with different chemicals, HT1080 cells were stained with the Prussian Blue Iron Stain Kit (Enhance with DAB, G1428, Solarbio, Beijing, China) according to the manufacturer’s instructions. In brief, cells were fixed with 4% paraformaldehyde for 20 min before being placed in a humidified chamber and stained with Perls working solution in the dark at 37 °C for 20 min. Cells were then washed three times with distilled water and then incubated with Incubation solution at 37 °C for 20 min in the dark. After being washed with PBS three times, the cells were stained with Enhanced solution in the dark at 37 °C for 20 min. Subsequently, the cells were stained with the Redyeing solution at room temperature in the dark for 5 min. After being washed with distilled water, the cells were photographed. Prussian-blue-stained positive cells exhibit yellowish brown to dark yellowish brown. Three fields of view with the same size were randomly captured for each sample.

### 2.8. 2,2-Diphenyl-1-Picrylhydrazyl (DPPH) Assay

The radical trapping activity of chemicals was assessed using the DPPH radical Scavenging Assay Kit (BC4755, Solarbio) following the manufacturer’s protocols. In the presence of antioxidants, the DPPH radical was scavenged, and the absorbance of its solution at 515 nm decreased. The ability of the sample to scavenge DPPH radicals was reflected by the degree of absorbance decrease. The working solution was prepared according to the instructions of the assay. A total of 190 μL of the working solution and 10 μL of different concentrations of drugs were mixed in a 96-well plate and incubated at room temperature in the dark for 30 min. The absorbance at 515 nm was detected with a Synergy H1 microplate reader (BioTek, Winooski, VT, USA). Different concentrations of Vitamin C were used as the DPPH standard. The DPPH radical scavenging ability of different concentrations of drugs was calculated based on the standard curve.

### 2.9. Kidney IRI

Eight- to ten-week male mice were intraperitoneally injected with Lip-1 (10 mg/kg) or silibinin (100 mg/kg) 24 h and 2 h before surgery. The renal IRI was induced by clamping the bilateral renal pedicle for 35 min, during which a heating table was used to maintain the body temperature of the mice. The mice in the Sham group experienced the same operation except clamping. All mice were sacrificed 24 h after surgery. Serum was collected for the detection of creatine and blood urea nitrogen. Renal tissue was fixed or stored for further analysis.

### 2.10. Hematoxylin–Eosin (H&E) Staining

Fresh tissues were fixed in 4% paraformaldehyde for 24 h. They were then dehydrated by gradient alcohol in a dehydrator. The tissues were then embedded in wax blocks. The embedded tissue was cut to a thickness of 4 μm. Paraffin sections were deparaffinized before staining with hematoxylin for nuclei and eosin for cytoplasm. After the staining was completed, the slices were dehydrated and sealed, and the image information was collected after drying. Sections were then scanned with panoramic scanning (3D HISTECH, Budapest, Hungary) to obtain high-definition images.

### 2.11. Terminal Deoxynucleotidyl Transferase-Mediated dUTP Nick End Labeling (TUNEL) Staining

Fresh tissues were fixed in 4% paraformaldehyde for 24 h. They were then dehydrated by gradient alcohol in a dehydrator. The tissues were then embedded in wax blocks. The embedded tissue was cut to a thickness of 4 μm. Paraffin sections were deparaffinized before staining with a DAB (SA-HRP) TUNEL Cell Apoptosis Detection Kit (Servicebio, Woburn, MA, USA) following the manufacturer’s guidance. After the staining was completed, the slices were dehydrated and sealed, and the image information was collected after drying. Sections were then scanned with panoramic scanning (3D HISTECH) to obtain high-definition images. Three fields of view with the same size were randomly selected for each sample and the number of dead cells was counted.

### 2.12. Q-PCR

Total RNA was purified from mice tissue using RNAiso Plus (Takara, Kyoto, Japan). A total of 1 μg of RNA was reverse transcribed into cDNA according to the instruction of HiScript III RT SuperMix for qPCR (Vazyme, Nanjing, China). The resulting cDNA was diluted five-fold with ultrapure water and used for assay. The experiment of real-time fluorescence quantitative PCR was carried out with 2× RealStar Power SYBR qPCR Mix (GenStar, San Francisco, CA, USA). The run method for qPCR was as follows: at the hold stage, 50 °C for 2 min, and 95 °C for 10 min. The PCR stage was performed at 95 °C for 15 s, and 60 °C for 1 min, for a total of 40 cycles. In the melt curve stage, 95 °C for 15 s, 60 °C for 1 min, and 95 °C for 15 s. The expression of GPX4, ACSL4, and prostaglandin-endoperoxide synthase 2 (PTGS2) genes in each tissue sample were then analyzed using the QuantStudio™ 6 Pro Real-Time PCR System (Thermo Fisher Scientific, Waltham, MA, USA). β-Actin mRNA was used as the internal reference. Relative Ct was used to detect gene expression levels. The PCR primers are as follows (Table 1):

### 2.13. Western Blot

Tissue samples were lysed in RIPA buffer. After centrifugation, the supernatant was collected and quantified by Pierce™ BCA Protein Assay Kits (Thermo Fisher Scientific) to yield protein concentrations. A total of 10 μg of protein from the sample was separated on a 12% SDS-PAGE gel before being transferred to a polyvinylidene fluoride membrane. They were blocked in 5% BSA for 1 h at room temperature and then washed 2–3 times with TBST. Membranes were trimmed and incubated with different primary antibodies overnight. Secondary antibodies were incubated at room temperature for 1 h after washing 4–5 times with TBST. The membranes were washed 4–5 times with TBST and exposed to Amersham ImageQuant 600 (GE Healthcare, Chicago, IL, USA). Western blotting was performed with Primary antibodies of GPX4 (Abcam, Cambridge, UK, ab125066), ACSL4/FACL4 (Abcam, ab155282), glyceraldehyde-3-phosphate dehydrogenase (GAPDH) (ABclonal, Woburn, MA, USA, AC033), and β-Actin (ABclonal, AC026). Secondary antibodies of rabbit IgG (CST, Danvers, MA, USA, 7074) and mouse IgG (CST, 7076) were used.

### 2.14. Malondialdehyde (MDA) Assay

Tissue MDA levels were measured with the MDA assay kit (Solarbio, BC0025). Briefly, the tissue samples were crushed in the MDA extraction reagent. After centrifugation, the supernatant was collected. The concentration of protein levels in the supernatant was measured with Pierce™ BCA Protein Assay Kits (Thermo Fisher Scientific). Then, we mixed the supernatant with MDA detection solution and incubated it at 100 °C for 60 min. After centrifugation, the supernatant was added to a 96-well plate and the absorbance at 450 nm, 532 nm, and 600 nm was measured by a Synergy H1 microplate reader (BioTek). The concentration of MDA was calculated using the following formula: MDA (nmol/mg prot) = 5 × (12.9 × (∆A532 − ∆A600) − 2.58 × ∆A450) ÷ Sample protein concentration.

### 2.15. GSH Assay

The GSH content was determined using GSH and GSSG Assay Kits (Beyotime, Shanghai, China, S0053) according to the manufacturer’s instructions. Briefly, 1.5 × 10^5^ HT1080 cells were seeded in a 12-well plate, and then treated with RSL3, silibinin, and Lip-1 for 4 h. The cells were then collected and washed with PBS. A total of 80 μL of Protein Removal Reagent M was mixed with the cell pellet. The samples were then frozen–thawed 3 times using liquid nitrogen and a 37 °C water bath. After 5 min of incubation in an ice bath and centrifugation at 10,000× *g* for 10 min at 4 °C, the total GSH level in the supernatant was measured. A total of 20 μL of the supernatant above was mixed with 4 μL GSH Removal Buffer and 0.8 μL GSH Removal Reagent, then incubated at 25 °C for 60 min. Following this, 10 μL of the supernatant before and after GSH removal were added to a 96-well plate, followed by the addition of 150 μL of the Total Glutathione Assay Buffer. The mixture was incubated at 25 °C for 5 min. Finally, 50 μL of NADPH (0.5 mg/mL) was added, and the absorbance at 412 nm was immediately measured by the Synergy H1 microplate reader (BioTek) at 5-min intervals six times. The GSH content was calculated using the formula:

GSH content = total GSH − 2 × GSSG

### 2.16. Statistics

The sample sizes were described in the figure legend and were determined for at least three biological replications. No exclusions of data were made that would significantly impact the results or conclusions. Animals with the same genotype and gender and similar age were randomly assigned to experimental groups. FlowJo version 10 was used for flow cytometry data analysis. GraphPad Prism 8 was used for statistical analysis. Results are shown as mean ± SD. In order to compare the groups based on a single factor, we employed the one-way analysis of variance (ANOVA) method. For the comparison between groups affected by two factors, we utilized the two-way analysis of variance (ANOVA) method. To account for the issue of multiple comparisons, we conducted a post-hoc test using either Tukey’s multiple comparison test or Bonferroni correction to determine significant differences among the groups (ns, not significant, * *p* < 0.05, ** *p* < 0.01, *** *p* < 0.001).

## 3. Results

### 3.1. Silibinin Inhibits Cell Ferroptosis

**Figure 1:**
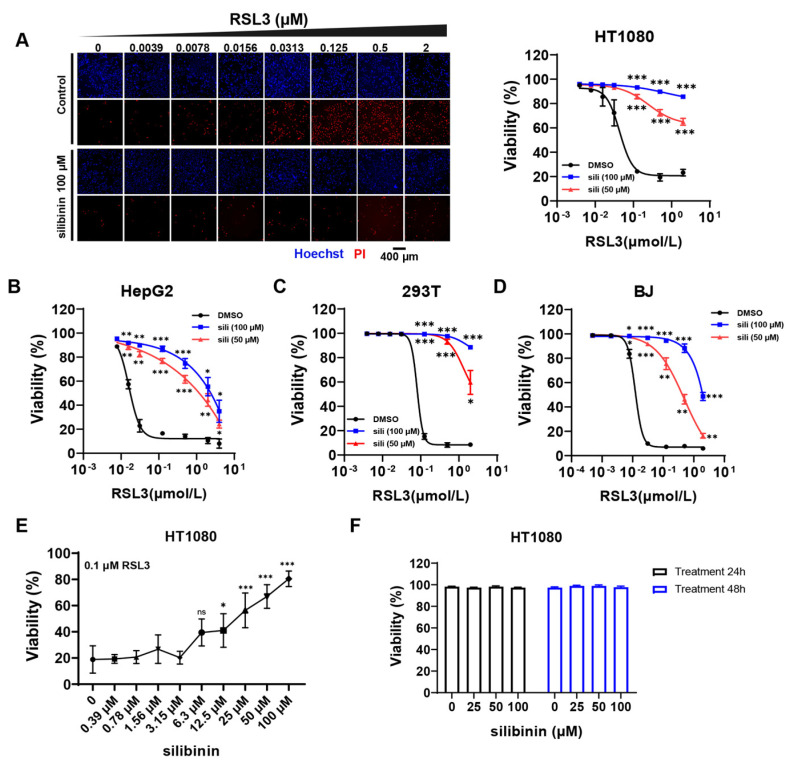
Silibinin inhibits cell ferroptosis. (**A**) Left panel: the representative images of HT1080 cells treated with different concentrations of RSL3 and 100 μM silibinin for 12 h following Hoechst and PI staining. Scale bar: 400 μm. Right panel: The statistical data of cell viability. The effect of silibinin under different concentrations of RSL3 were compared with the corresponding DMSO group. (**B**) The viability of HepG2 cells treated with different concentrations of RSL3 for 24 h with or without silibinin (sili). The effect of silibinin under different concentrations of RSL3 were compared with the corresponding DMSO group. (**C**) The viability of 293T cells treated with different concentrations of RSL3 for 24 h with or without silibinin (sili). The effect of silibinin under different concentrations of RSL3 were compared with the corresponding DMSO group. (**D**) The viability of BJ cells treated with different concentrations of RSL3 for 24 h with or without silibinin (sili). The effect of silibinin under different concentrations of RSL3 were compared with the corresponding DMSO group. (**E**) The viability of HT1080 cells treated with 0.1 μM RSL3 and different concentrations of silibinin for 24 h. All intergroup comparisons were compared with the control (silibinin 0 μM) group. (**F**) The viability of HT1080 cells treated with different concentrations of silibinin for 24 h or 48 h. The values were presented as mean ± SD of three independent biological replications; ns, no significance; * *p* < 0.05, ** *p* < 0.01, *** *p* < 0.001.

Among lipophilic natural compounds with antioxidant activity, silibinin has been extensively utilized in clinical applications and healthcare. Moreover, silibinin has demonstrated effective mitigation of liver injury [19], which is implicated in ferroptosis [18]. To investigate the effect of silibinin on cell ferroptosis, HT1080 cells were treated with different concentrations of RSL3, which induces ferroptosis through inhibiting GPX4 [5]. We found that the viability of cells gradually decreased with the increase in RSL3 concentration, while silibinin effectively reduced cell ferroptosis (Figure 1A). Similarly, RSL3-induced cell death of HepG2, 293T, and BJ was also inhibited by silibinin (Figure 1B–D). In addition, ferroptosis induced by erastin, a system Xc^-^ inhibitor that blocks GSH synthesis [1], in all of these four cell lines was eliminated by silibinin as well (Figure S1). Furthermore, the ferroptosis elimination effect of silibinin in HT1080 cells was found to be concentration-dependent (Figure 1E). Finally, to test the toxicity of silibinin, HT1080 cells were treated with different concentrations of silibinin. We found that cell viability was higher than 96% after a 48 h treatment with 25–100 μM silibinin (Figure 1F). The above results suggest that silibinin is a safe and effective ferroptosis inhibitor.

### 3.2. Silibinin Inhibits Ferroptosis through Antioxidant Activity

**Figure 2:**
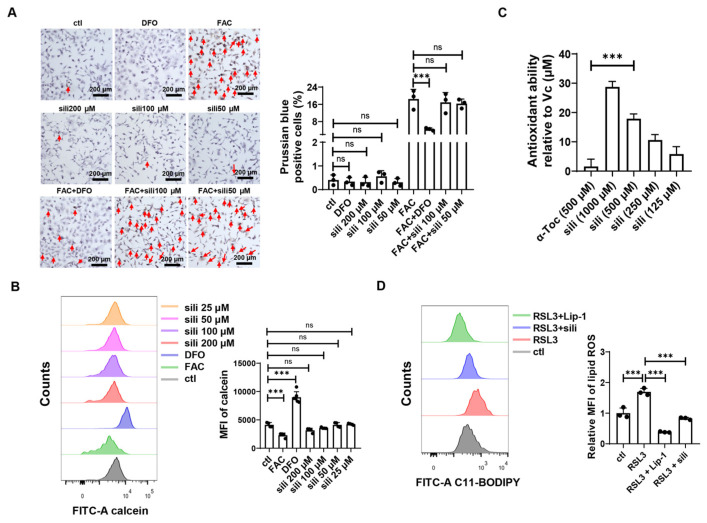
Silibinin inhibits ferroptosis through its antioxidant ability. (**A**) Left panel: representative images of Prussian blue staining of HT1080 cells treated with 100 μM ammonium iron (Ⅲ) citrate (FAC), 100 μM DFO, or various concentrations of silibinin. Scale bar: 200 μm. Prussian blue staining-positive cells are marked with red arrows. Right panel: the statistical data of Prussian-blue-positive cells. Three independent replications were performed and three random fields of view with the same size from each replication were quantified. (**B**) Left panel: the representative flow cytometry results of Calcein-AM staining detected by FITC channel in HT1080 cells treated with Deferoxamine (DFO, 100 μM), ammonium iron (Ⅲ) citrate (FAC, 100 μM), or different concentrations of silibinin for 24 h. Right panel: the statistical data of MFI of Calcein-AM staining. (**C**) Antioxidant ability of α-Toc (500 μM) and different concentrations of silibinin (sili) relative to Vc determined by DPPH assays. (**D**) Left panel: the representative result of lipid peroxidation was measured by C11-BODIPY with FITC channel in HT1080 cells post 4 h treatment with RSL3 (125 nM), Liproxstatin-1(Lip-1, 1 μM), silibinin (sili, 100 μM). Right panel: the statistical data of MFI of lipid ROS. The values were presented as mean ± SD of three independent biological replications; ns, no significance; *** *p* < 0.001.

Next, we explored the mechanism by which silibinin inhibits ferroptosis. Previous studies have reported that silibinin may affect iron levels in cells [20,21,22]. To examine the influence of ferroptosis inducers and silibinin on the total iron level in HT1080 cells, Prussian blue staining was performed. The staining in the untreated cells was rather subtle, indicating low iron levels in these cells. Neither RSL3 nor erastin affected the total iron level (Figure S2A,B), while Ammonium iron (Ⅲ) citrate (FAC) significantly augmented iron accumulation, which was countered by DFO (Figure 2A and Figure S2A,B). However, FAC-induced iron augmentation was not affected by the treatment of various concentrations of silibinin (Figure 2A). These results suggest that silibinin does not affect the total iron levels. To examine if silibinin inhibited ferroptosis by affecting the labile iron pool, we stained cells with Calcein-AM, of which fluorescence is quenched upon binding to labile iron. The fluorescence intensity of Calcein in HT1080 cells treated with FAC was reduced, suggesting an increase in the level of the labile iron pool. The fluorescence intensity of Calcein increased in cells treated with DFO but not different concentrations of silibinin, indicating that silibinin did not affect the labile iron pool (Figure 2B). Next, considering the antioxidant capacity of silibinin [23], we tested its radical trapping activity in vitro through the DPPH assay. Silibinin showed a concentration-dependent and stronger antioxidant ability than α-Toc at the same concentration (Figure 2C). Moreover, silibinin mitigated lipid peroxidation in RSL3-treated cells as Lip-1, a widely used ferroptosis inhibitor in vitro and in vivo (Figure 2D). Next, we detected the glutathione (GSH) level (Figure S2C) and the expression of antioxidant genes including CAT and SOD1. The results showed that they were not affected by silibinin or Lip-1 (Figure S2D). In addition, the expression of genes in the lipid peroxidation scavenging system including GPX4, SLC7A11, FSP1, GCH1, and DHODH was not influenced by silibinin either (Figure S2E). Hence, silibinin inhibits cell ferroptosis through its antioxidant activity instead of affecting iron levels or antioxidant systems.

### 3.3. Silibinin Attenuates Ferroptosis-Induced Liver Injury in GPX4-Deficient Mice

**Figure 3:**
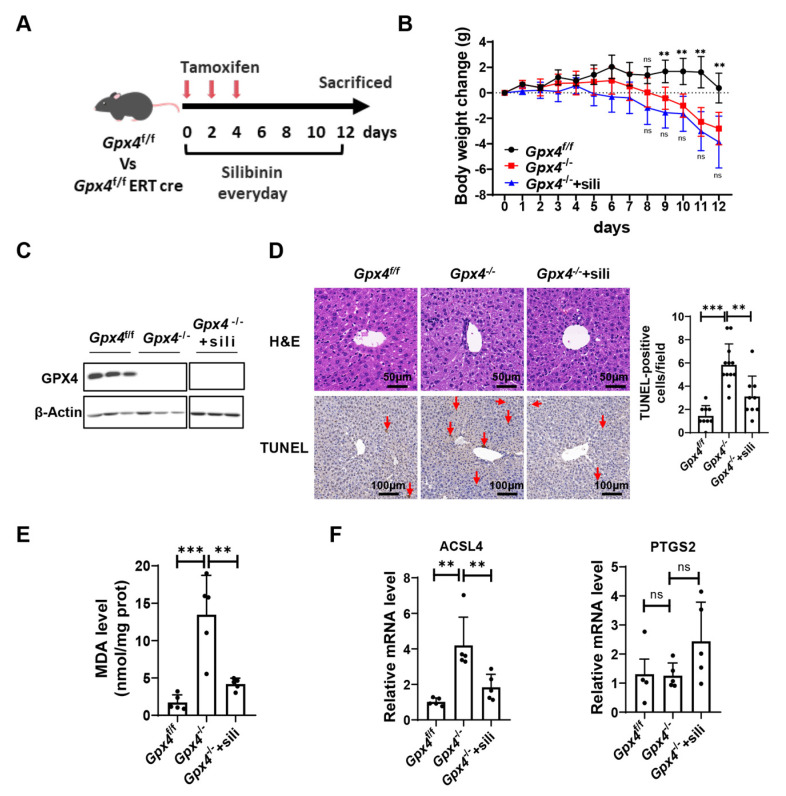
Silibinin attenuates GPX4-deficiency-induced ferroptosis in liver. (**A**) The schematic of experiments in vivo. *Gpx4*^flox/flox^ (*Gpx4*^f/f^) mice or *Gpx4*^flox/flox^ ERT-Cre (*Gpx4*^−/−^) mice were injected with tamoxifen (100 mg/kg) every other day three times. At the same time, *Gpx4*^−/−^ mice were treated with silibinin (sili, 100 mg/kg) (*Gpx4*^−/−^+sili) every day until sacrificed. (**B**) The changes in body weight of *Gpx4*^f/f^ and *Gpx4*^−/−^ mice treated with or without silibinin. All comparisons between groups were compared with the *Gpx4*^−/−^ group on the same day; n = 5 mice. (**C**) The protein levels of GPX4 in the liver of the indicative mice were measured by Western blot. (**D**) Left panel: representative images of H&E staining of liver sections in the indicative mice. Scale bar: 50 μm; n = 5 mice. Representative images of TUNEL staining of liver sections in the indicative mice. Scale bar: 100 μm. TUNEL-positive cells are marked with red arrows; n = 3–4 mice. Right panel: the statistical data of TUNEL staining; n = 3–4 mice. Three random fields of view with the same size were selected for each sample. (**E**) Malondialdehyde (MDA) levels in the liver of the indicative mice were measured; n = 5 mice. (**F**) mRNA levels of PTGS2 and ACSL4 in the liver of the indicative mice were measured; n = 4–5 mice. The values were presented as mean ± SD; ns, no significance; ** *p* < 0.01, *** *p* < 0.001.

Ferroptosis has been increasingly recognized as a promising target for various diseases such as neurodegeneration, acute liver injury, and IRI [24]. Next, we investigated the effect of silibinin on ferroptosis in vivo. Silibinin was administrated in *Gpx4*^flox/flox^ ERT-Cre mice, which were treated with tamoxifen to induce *Gpx4* gene knockout (Figure 3A). We found that GPX4-deficient mice (*Gpx4*^−/−^ mice) gradually lost body weight, which was not affected by silibinin administration (Figure 3B).

Next, we monitored the effect of silibinin on different organs in the GPX4-deficient mice. Firstly, the protein level of GPX4 in the liver was verified (Figure 3C). H&E staining showed a disrupted structure of the hepatic lobule and unclear intercellular space between hepatocytes in *Gpx4*^−/−^ mice, while treatment with silibinin significantly ameliorated these phenotypes (Figure 3D). TUNEL staining revealed an increased number of dead cells in the liver of *Gpx4*^−/−^ mice compared with *Gpx4*^flox/flox^ mice. With silibinin treatment, TUNEL-positive cells were significantly reduced (Figure 3D). The increased level of MDA, a final product of lipid peroxidation and a biomarker of ferroptosis, indicates that hepatocytes underwent ferroptosis in GPX4-deficient mice (Figure 3E). Strikingly, silibinin alleviated liver injury and reduced MDA levels in GPX4-deficient mice (Figure 3D,E). The increased expression of ACSL4 and PTGS2 were regarded as biomarkers of ferroptosis [25]. The deficiency of GPX4 resulted in an elevated expression of ACSL4 in the liver, which was inhibited by silibinin administration. However, we did not observe a significant difference in PTGS2 expression levels between GPX4-deficient mice with or without silibinin treatment (Figure 3F). In summary, silibinin attenuates ferroptosis-induced liver injury in GPX4-deficient mice.

### 3.4. Silibinin Attenuates Ferroptosis in the Kidney of GPX4-Deficient Mice

**Figure 4:**
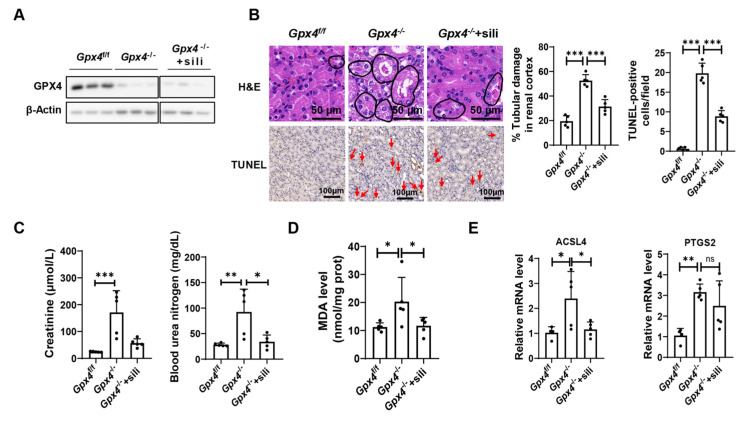
Silibinin inhibits GPX4 deficiency-induced ferroptosis in the kidney. (**A**) The protein levels of GPX4 in the kidney of the indicative mice were measured by Western blot. (**B**) Left panel: the representative images of H&E staining and TUNEL staining of kidney sections in the indicative mice. Scale bar: 50 μm in H&E staining and 100 μm in TUNEL staining. The damaged tubules are marked by black circles. TUNEL-positive cells are marked with red arrows. Middle panel: The statistical data of H&E staining; n = 5 mice. Right panel: The statistical data of TUNEL staining. Three random fields of view with the same size were selected for each sample; n = 5 mice. (**C**) Serum levels of creatinine and blood urea nitrogen were measured; n = 5 mice. (**D**) MDA levels in the kidney of the indicative mice were measured; n = 5 mice. (**E**) mRNA levels of PTGS2 and ACSL4 in the kidney of the indicative mice were measured; n = 4–5 mice. The values were presented as mean ± SD; ns, no significance; * *p* < 0.05, ** *p* < 0.01, *** *p* < 0.001.

Silibinin and silymarin are widely used to treat chronic and acute liver disease [26]. However, their effects on other organs remain unclear. Next, we examined tissue injury in the kidney of GPX4-deficient mice. The results showed that GPX4 is deleted in the kidney of *Gpx4*^−/−^ mice (Figure 4A). The results of TUNEL staining and H&E staining of the kidney showed that GPX4 deficiency resulted in cell death and tubular damage in the renal cortex, which was attenuated by silibinin treatment (Figure 4B). Furthermore, significant increases in the serum levels of creatinine and blood urea nitrogen in the *Gpx4*^−/−^ mice compared with GPX4-sufficient mice (*Gpx4*^f/f^ mice) were found (Figure 4C), which indicated that GPX4 deletion results in severe renal injury. Strikingly, the levels of serum creatinine and blood urea nitrogen significantly decreased after silibinin administration (Figure 4C). To evaluate ferroptosis in the kidney of mice, the MDA level was measured. The results showed that GPX4 deletion resulted in an increased level of MDA in the kidney, and silibinin treatment reversed this phenotype (Figure 4D). Furthermore, GPX4 deletion elevated the expression of ACSL4 and PTGS2. However, after silibinin treatment, the expression of ACSL4 in the kidney decreased (Figure 4E). These results suggest that silibinin inhibits renal ferroptosis in GPX4-deficient mice.

Besides the liver and kidney, the effect of silibinin on the lung and heart in GPX4-deficient mice was evaluated. The protein level of GPX4 in the lung and heart of *Gpx4*^−/−^ mice was verified (Figure S3A,B). However, the tissue morphology indicated through H&E staining, the MDA level, and the expression of ACSL4 and PTGS2 in the heart and lung proved to be intact after GPX4 deletion, demonstrating that GPX4 deletion did not cause ferroptosis and tissue injury in the heart and lung (Figure S3C–I).

### 3.5. Silibinin Alleviates Renal Ischemia-Reperfusion Injury

**Figure 5:**
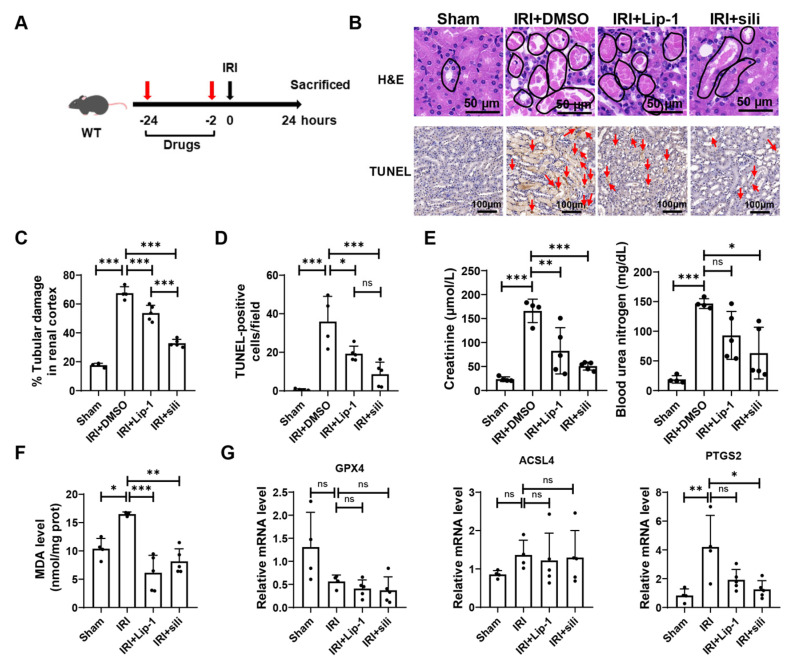
Silibinin rescues renal ischemia-reperfusion injury. (**A**) The schematic of renal ischemia-reperfusion. A total of 24 h and 2 h before surgery, the mice were intraperitoneally injected with Lip-1 (10 mg/kg) or silibinin (sili, 100 mg/kg). Mice were sacrificed 24 h after surgery. (**B**) The representative images of H&E staining and TUNEL staining of kidney sections in the indicative mice. Scale bar: 50 μm in H&E staining and 100 μm in TUNEL staining. The damaged tubules are marked by black circles. TUNEL-positive cells are marked with red arrows. n = 4–5 mice. (**C**) The statistical data of H&E staining; n = 4–5 mice. Three random fields of view with the same size were selected for each sample. (**D**) The statistical data of TUNEL staining; n = 4–5 mice. Three random fields of view with the same size were selected for each sample. (**E**) Serum levels of creatinine and blood urea nitrogen were measured; n = 4–5 mice. (**F**) MDA levels in the kidney of the indicative mice were measured; n = 4–5 mice. (**G**) mRNA levels of GPX4, ACSL4, and PTGS2 in the kidney of the indicative mice were measured; n = 4–5 mice. The values were presented as mean ± SD; ns, no significance; * *p* < 0.05, ** *p* < 0.01, *** *p* < 0.001.

Next, we tested the effect of silibinin on ferroptosis-related disease, among which renal IRI is well studied (Figure 5A). Consistent with a previous study [27], the renal tubules appeared obvious lesions post renal ischemia-reperfusion, which was partially rescued by Lip-1 pretreatment. Strikingly, silibinin presented a significant protective effect on the morphology of renal tissue, even better than Lip-1. Renal ischemia-reperfusion also caused severe cell death as indicated by a significant increase in TUNEL-positive cells in the kidney. Treatment with silibinin or Lip-1 significantly diminished the number of TUNEL-positive cells (Figure 5B–D). In addition, the levels of creatinine and blood urea nitrogen in serum were highly elevated after renal ischemia-reperfusion, indicating severe injury in renal tissues. However, these injuries were significantly reduced in mice treated with Lip-1 or silibinin (Figure 5E). Furthermore, ischemia-reperfusion resulted in a significant increase in the MDA level in the kidney, which was prevented by Lip-1 or silibinin (Figure 5F). The expression level of GPX4 was found to be significantly decreased while that of both ACSL4 and PTGS2 increased in the kidney after ischemia-reperfusion. Silibinin obviously downregulated the expression of PTGS2 (Figure 5G). In conclusion, silibinin is an anti-ferroptosis chemical, which effectively inhibits renal injury induced by ischemia-reperfusion.

## 4. Discussion

Because of its critical role in multiple physiological and pathological processes, ferroptosis has been increasingly studied in recent years. Especially, the identification and development of drugs that inhibit or activate ferroptosis, which are potentials for the therapy of multiple diseases such as cancer, neurodegeneration, and other ferroptosis-related tissue injuries, are receiving more and more attention [28]. Here, we revealed that silibinin, a widely used healthcare chemical, inhibits ferroptosis induced by either RSL3 or erastin in different types of cells. In vivo, silibinin treatment effectively alleviates ferroptosis-related injuries in the liver and kidney of GPX4-deficient mice. Moreover, renal IRI, in which ferroptosis has been reported to be critical, is ameliorated by silibinin. Thus, our study suggests that silibinin inhibits cell ferroptosis and ferroptosis-related tissue injuries. Consistently, a recent report showed that silibinin inhibits ethanol-induced ferroptosis in the liver cell line [29].

Previous studies have reported that silibinin inhibits ferroptosis accompanied by decreased levels of total iron in rat insulinoma INS-1 cells treated with palmitic acid (PA) plus high glucose (HG) and liver cell lines treated with ethanol or acetaldehyde [29,30]. Here, we found that RSL3 and erastin induce ferroptosis without affecting the total iron level in HT1080 cells. Moreover, silibinin inhibits ferroptosis through its antioxidant activity rather than affecting the total or labile iron pool in cells. Silibinin has been well-proved to be safe and has been widely used for the treatment of liver diseases [13]. However, the effects of silibinin on other tissues or organs are underestimated because of its poor solubility and bioavailability in vivo [14]. Here, we found that silibinin alleviates ferroptosis-related injuries in both the liver and kidney. Therefore, improving the bioavailability of silibinin through a suitable delivery system may be promising to cure multiple diseases in organs besides the liver [31,32].

Ferroptosis-related injuries such as ischemia-reperfusion injury, involve massive cell death and inflammation, leading to severe diseases including ischemic heart disease, brain stock, and organ damages during transplantation [12]. Thus, ferroptosis inhibitors are promising in the therapy for these intractable diseases. However, the known ferroptosis inhibitors such as iron chelators are limited by their off-target effect and toxicity, while hydrophobic antioxidants may vary widely in different contexts [11,12]. Here, our results showed that silibinin exhibits a stronger antioxidant capacity than α-Toc, a classical hydrophobic radical-trapping antioxidant, directly ameliorating lipid peroxidation [11]. Therefore, silibinin, as a safe and effective ferroptosis inhibitor in vivo, has broad application prospects.

Here, silibinin was administered either before ischemia-reperfusion or together with GPX4 deficiency induction in vivo. To demonstrate the efficacy of silibinin in a more clinically translational situation, an after-treatment of the silibinin experiment should be conducted in future studies.

## 5. Conclusions

As demonstrated by this study, we found that silibinin effectively inhibits ferroptosis induced by different inducers. Meanwhile, silibinin alleviates the organ injuries caused by GPX4 deletion and renal ischemia-reperfusion injury in mice. Our findings suggest that silibinin is a potential chemical for treating ferroptosis-related diseases.

## Figures and Tables

**Table 1:** List of PCR primers.

The PCR Primers		Sequence
Mouse-GPX4	Forward	CCTCTGCTGCAAGAGCCTCCC
Mouse-GPX4	Reverse	CTTATCCAGGCAGACCATGTGC
Mouse-ACSL4	Forward	CCTTTGGCTCATGTGCTGGAAC
Mouse-ACSL4	Reverse	GCCATAAGTGTGGGTTTCAGTAC
Mouse-PTGS2	Forward	GCGACATACTCAAGCAGGAGCA
Mouse-PTGS2	Reverse	AGTGGTAACCGCTCAGGTGTTG
Mouse-β-Actin	Forward	GGCTGTATTCCCCTCCATCG
Mouse-β-Actin	Reverse	CCAGTTGGTAACAATGCCATGT
Human-GPX4	Forward	GAGGCAAGACCGAAGTAAACTAC
Human-GPX4	Reverse	CCGAACTGGTTACACGGGAA
Human-CAT	Forward	TGGAGCTGGTAACCCAGTAGG
Human-CAT	Reverse	CCTTTGCCTTGGAGTATTTGGTA
Human-FSP1	Forward	GATGAGCAACTTGGACAGCAA
Human-FSP1	Reverse	CTGGGCTGCTTATCTGGGAAG
Human-GCH1	Forward	GCCATGCAGTTCTTCACCAAGG
Human-GCH1	Reverse	ATGGAACCAAGTGATGCTCACAC
Human-SLC7A11	Forward	TCCTGCTTTGGCTCCATGAACG
Human-SLC7A11	Reverse	AGAGGAGTGTGCTTGCGGACAT
Human-DHODH	Forward	GAGGACATTGCCAGTGTGGTCA
Human-DHODH	Reverse	TTCCCACTCAGCCCTCCTGTTT
Human-SOD1	Forward	GGTGGGCCAAAGGATGAAGAG
Human-SOD1	Reverse	CCACAAGCCAAACGACTTCC
Human-β-Actin	Forward	CATGTACGTTGCTATCCAGGC
Human-β-Actin	Reverse	CTCCTTAATGTCACGCACGAT

## Data Availability

Data are contained within the article and supplementary materials.

## Supplementary Materials

The following supporting information can be downloaded at https://www.mdpi.com/article/10.3390/antiox12122119/s1, Figure S1: Silibinin inhibits ferroptosis in different kinds of cells induced by erastin; Figure S2: Silibinin does not affect total iron levels, GSH levels and antioxidant gene expression; Figure S3: GPX4 deletion does not induce significant lung and heart injury in mice.
