# Supplementary material for: Silibinin Inhibits Cell Ferroptosis and Ferroptosis-Related Tissue Injuries
Source: Antioxidants (Basel). 2023 Dec 15;12(12):2119. doi: 10.3390/antiox12122119 (PMC10740598; doi:10.3390/antiox12122119)
Supplement: Supplementary file 1 [file antioxidants-12-02119-s001.zip › antioxidants-2656651-supplementary.pdf]

antioxidants

MDPI

Supplementary figure

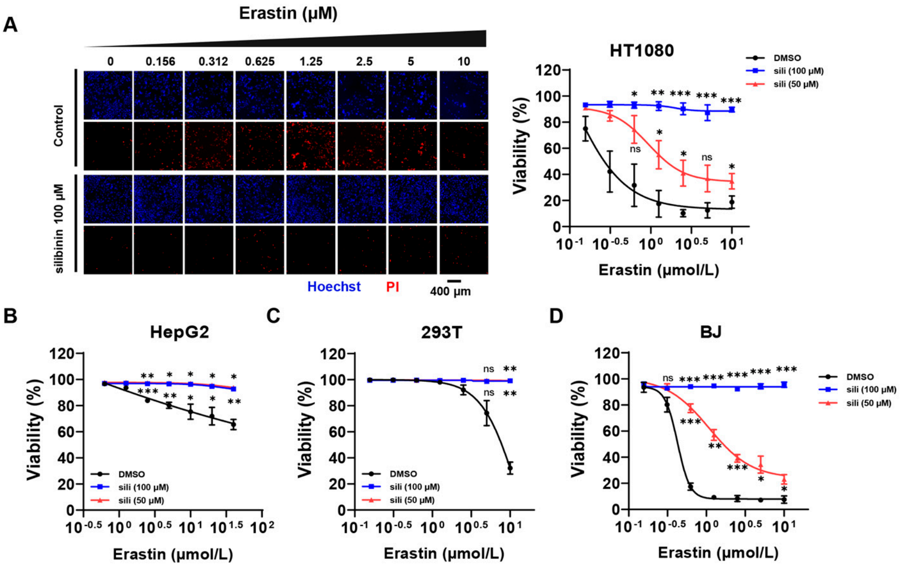

Figure S1. Silibinin inhibits ferroptosis in different kinds of cells induced by erastin. (A) Left panel: the representative images of HT1080 cells treated with different concentrations of erastin and silibinin (sili) for 24 h following Hoechst and PI staining. Scale bar: 400 μm. Right panel: The statistical data of cell viability. (B) The viability of HepG2 cells treated with different concentrations of Erastin for 24 h with or without silibinin (sili). (C) The viability of 293T cells treated with different concentrations of Erastin for 24 h with or without silibinin (sili). (D) The viability of BJ cells treated with different concentrations of Erastin for 24 h with or without silibinin (sili). The effect of silibinin under different concentration of RSL3 were compared with the corresponding DMSO group. All comparisons between groups were compared with the DMSO group. The values were presented as mean ± SD. *P < 0.05, **P < 0.01, *** P < 0.001.

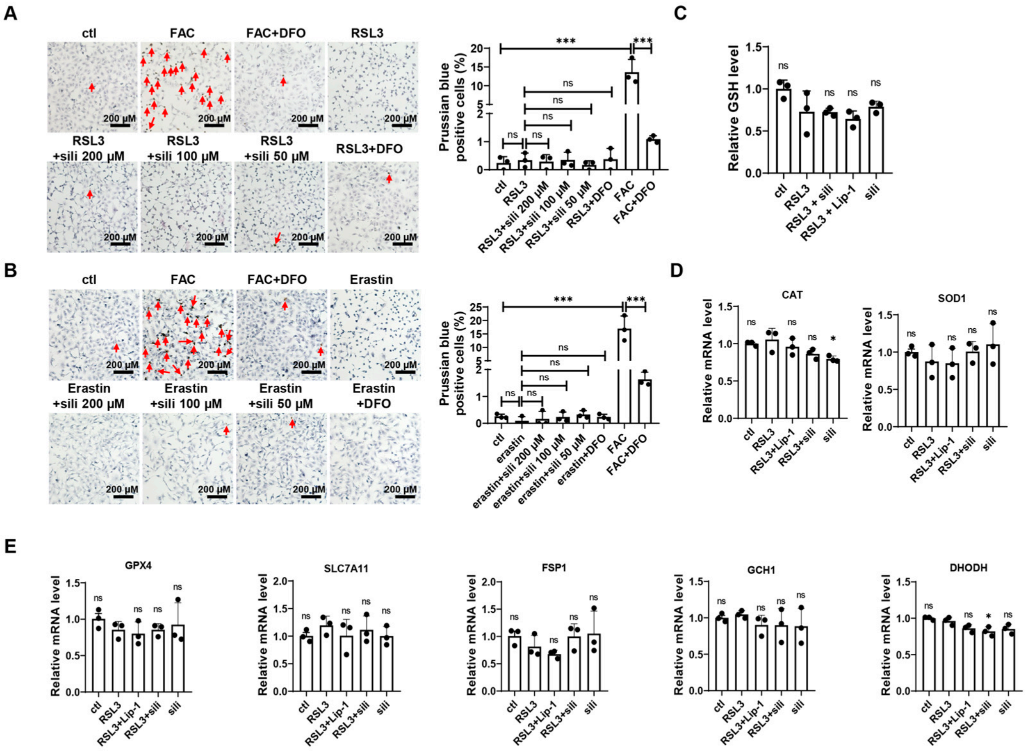

Figure S2. Silibinin does not affect total iron levels, GSH levels and antioxidant gene expression. (A) Left panel: representative images of Prussian blue staining of HT1080 treated with 100 μM ammonium iron (III) citrate (FAC), 100 μM DFO, 125 nM RSL3, or various concentrations of silibinin for 4 h. Scale bar: 200 μm. Prussian blue staining-positive cells are marked with red arrows. Right panel: the statistical data of Prussian blue positive cells. Three independent replications were performed and three random fields of view with the same size from each replication were quantified. (B) Left panel: representative images of Prussian blue staining of HT1080 treated with 100 μM ammonium iron (III) citrate (FAC), 100 μM DFO, 10 μM erastin, or various concentrations of erastin for 8 h. Scale bar: 200 μm. Prussian blue staining-positive cells are marked with red arrows. Right panel: the statistical data of Prussian blue positive cells. Three independent replications were performed and three random fields of view with the same size from each replication were quantified. (C) The GSH level in HT1080 cells treated with RSL3 (125 nM), RSL3 (125 nM) +Lip-1 (1 μM), or RSL3 (125 nM) +silibinin (sili, 100 μM) was measured. All comparisons between groups were compared with the RSL3 group. (D) The expression level of antioxidant genes including CAT and SOD1 in HT1080 cells treated with RSL3 (125 nM), RSL3 (125 nM) +Lip-1 (1 μM), or RSL3 (125 nM) + silibinin (sili, 100 μM) were measured. All comparisons between groups were compared with the RSL3 group. (E) The expression of genes in the lipid peroxidation scavenging system including GPX4, SLC7A11, FSP1, GCH1, and DHODH in HT1080 cells treated with RSL3 (125 nM), RSL3 (125 nM) +Lip-1 (1 μM), or RSL3 (125 nM) + silibinin (sili, 100 μM) were measured. All comparisons between groups were compared with the RSL3 group. The values were presented as mean ± SD. ns, no significance, *P < 0.05, ***P < 0.001.

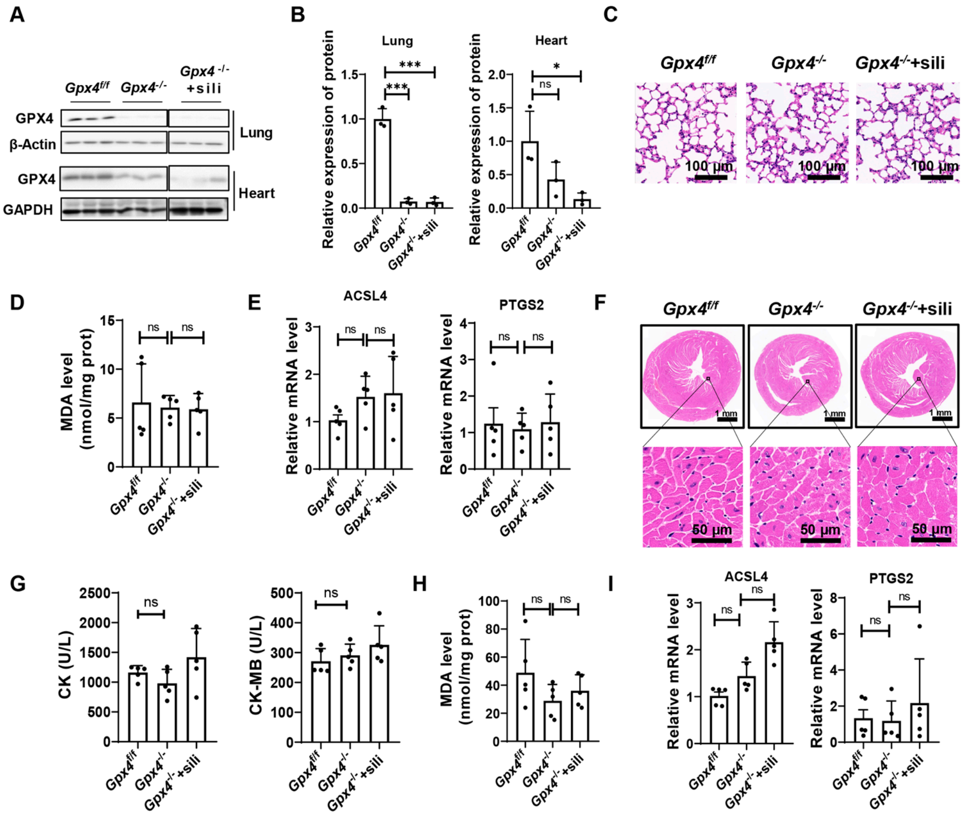

Figure S3. GPX4 deletion does not induce significant lung and heart injury in mice. (A)The protein levels of GPX4 in the lung and heart of the indicative mice were measured by Western blot. (B)The statistical data of protein expression. (C)Representative images of H&E staining of lung sections in the indicative mice. n = 5 mice. Scale bar: 100 μm. (D)MDA levels in the lung of the indicative mice were measured. n = 5 mice. (E)mRNA levels of PTGS2 and ACSL4 in the lung of the indicative mice were measured. n = 5 mice. (F)Representative images of H&E staining of heart section in the indicative mice. n = 5 mice. (G)Serum levels of Creatine Kinase (CK) and Creatine Kinase Isoenzymes (CK-MB) were measured. n = 5 mice. (H)MDA levels in the heart of the indicative mice were measured. n = 5 mice. (I)mRNA levels of PTGS2 and ACSL4 in the heart of the indicative mice were measured. n = 5 mice. The values were presented as mean ± SD. ns, no significance, *P < 0.05.
